# Clinicopathological features and EBV infection status of lymphoma in children and adolescents in South China: a retrospective study of 662 cases

**DOI:** 10.1186/s13000-018-0693-0

**Published:** 2018-02-27

**Authors:** Changfei Qin, Yuhua Huang, Yanfen Feng, Min Li, Na Guo, Huilan Rao

**Affiliations:** 1 State Key Laboratory of Oncology in South China, Collaborative Innovation Center for Cancer Medicine, Guangzhou, Guangdong 510060 People’s Republic of China; 2 Department of Pathology, Sun Yat-sen University Cancer Center, Guangzhou, Guangdong 510060 People’s Republic of China

**Keywords:** Lymphoma, Epstein-Barr virus (EBV), Children and Adolescents, South China

## Abstract

**Background:**

The clinicopathological features and Epstein-Barr virus (EBV) infection status of lymphoma in children and adolescents in South China is under-researched. South China is a well-known high-incidence area of EBV-associated nasopharyngeal carcinoma.

**Methods:**

A cohort of 662 consecutive children and adolescents’ lymphomas was retrospectively analyzed and Epstein-Barr virus encoded RNAs (EBERs) in situ hybridization was performed to detect the EBV infection.

**Results:**

The majority (501/662, 75.7%) of lymphomas in children and adolescents was Non-Hodgkin lymphoma (NHL). One hundred sixty one cases (24.3%) were Hodgkin lymphoma (HL). Of the NHL, precursor cell lymphoma, mature B-cell lymphoma and peripheral T/NK-cell lymphoma accounted for 32.0%, 41.1% and 26.9% respectively. The five common subtypes were lymphoblastic lymphoma (32.0%), Burkitt lymphoma (BL) (21.0%), anaplastic large-cell lymphoma (ALCL) (14.2%), diffuse large B-cell lymphoma (DLBCL) (13.8%) and extranodal NK/T-cell lymphoma, nasal type (ENKTCL) (6.2%). EBV infection was detected in 58.9% classical Hodgkin lymphomas (CHLs), 21.4% mature B-cell lymphomas and 52.4% peripheral T/NK-cell lymphomas. Moreover, EBV was associated with high grade NHL including ENKTCL (100.0%), BL (30.5%) and DLBCL (17.6%).

**Conclusion:**

The high proportion of peripheral T/NK-cell lymphomas in children and adolescents in South China are presented in this study and compared to western countries due to the high percentage of ENKTCL. ENKTCL is firmly associated with EBV infection, while more than half of HL, a portion of BL and DLBCL are related to EBV infection. This study conclusively demonstrates that EBV infection is more prevalent in children and adolescents with lymphomas in South China compared to western countries.

**Electronic supplementary material:**

The online version of this article (10.1186/s13000-018-0693-0) contains supplementary material, which is available to authorized users.

## Background

Lymphoid neoplasms are the most common neoplasms in children and adolescents worldwide and represent a major cause of tumor death in China [1, 2]. Generally, malignant lymphomas have been grouped into two categories: Hodgkin’s lymphoma (HL) and Non-Hodgkin’s lymphoma (NHL). HL is one of the most curable forms of cancer with an estimated 5-year survival rate exceeding 98% [3]. However, NHL is more complex and has been classified by the World Health Organization (WHO) based on phenotype (B vs T vs NK-cell lineage) and differentiation (i.e. Precursor vs mature) [4].

Most reports of NHLs in children and adolescents are high grade lymphomas, including Burkitt’s lymphoma (BL), diffuse large B-cell lymphoma (DLBCL), lymphoblastic lymphoma (LBL) and anaplastic large cell lymphoma (ALCL) [5–7]. The occurrence and clinicopathologic features of low grade lymphomas in children and adolescents have not been well characterized and are not up to date.

It is well established that Epstein-Barr virus (EBV) plays an important role in some subtypes of lymphomas, such as CHL, extranodal NK/T cell lymphoma (ENKTCL) and BL [8–10]. In addition, South China is a high-incidence area of EBV-associated nasopharyngeal carcinoma, thus the clinicopathological features and Epstein-Barr virus (EBV) infection status of lymphoma in children and adolescents in South China is worthy of investigation. In this study, a cohort of 662 consecutive lymphomas in children and adolescents were retrospectively analyzed in which Epstein-Barr virus encoded RNAs (EBERs) in situ hybridization was performed to detect EBV infection.

## Methods

### Patient selection

A total of 662 cases of lymphoma in children and adolescents (≦20 years old) were obtained retrospectively from the Department of Pathology at Sun Yat-sen University Cancer Center during the period of January 2010 to January 2014. A diagnostic criterion was established according to the 4th revised edition of the WHO classification of Tumours of Haematopoietic and Lymphoid Tissues [11]. Diagnosis was confirmed by two hematopathologists.

### Clinical characteristics analysis

The clinical data, including gender, age, and tumor localization (biopsy site) were analyzed. Slides and images were reviewed including biopsy of the lymph node or mass, computed tomography (CT) scan of the chest, abdominopelvic ultraso-nography, and bone marrow biopsies in selected cases.

### Distribution of lymphoma subtypes and EBV detection

The data were available on a standard panel of immunochemistry stains using antibody anti-CD20, CD79, PAX-5, CD2, CD3, CD4, CD5, CD7, CD8, CD30, CD56, CD15, ALK (anaplastic large cell lymphoma kinase), and on a fluorescence in situ hybridization (FISH) for the chromosomal translocation juxtaposing the c-myc oncogene and immunoglobulin locus regulatory element of it (8;14) in the dedicated diagnosis of BL [12]. IgH and/or TCR gene rearrangement detection using polymerase chain reaction (PCR) was performed in the diagnosis of uncertain cases by routine histopathologic and immunophenotypic evaluation. Subtype distribution of lymphoma was analyzed according to the WHO criteria. EBV was detected using the EBV Probe In Situ Hybridization Kit (TRIPLEX INTERNATIONAL BIOSCIENCES, CHINA, CO. LTD).

### Statistical analysis

Data were statistically described using means±standard deviation (SD), range, frequencies (number of cases) and percentages when appropriate. All statistical calculations were carried out using the Statistical Package for the Social Sciences software (SPSS), version 19.

## Results

### Clinical features

The median age was 13 years old and the ratio of male to female was 1.68:1. Most lymphomas showed a male predominance including classical Hodgkin’s lymphoma (CHL) (M/F = 1.68), BL (M/F = 5.56), ALCL (M/F = 1.84) and LBCL (M/F = 1.47), while ENKTCL showed a slight female predominance (M/F = 0.82) (Additional file 1: Table S1 and Table 1).

**Table 1:** Distribution of HL subtypes in children and adolescents in South China (*Ν* = 161)

Lymphoid Neoplasms	No.of cases	% of HLs	M:F Ratio	% of EBERs +
HL	161	100.0	1.6	54.6 (53/97)
CHL, NS	80	49.7	1.9	44.7 (21/47)
CHL, MC	47	29.2	1.9	76.9 (20/26)
CHL, LR	21	13.0	0.8	68.8 (11/16)
CHL, LD	1	0.6	#	100.0 (1/1)
NLPHL	12	7.5	1.0	0.0 (0/7)

*CHL-MC* mixed cellularity classical Hodgkin lymphoma, *CHL-NS* nodular sclerosis classical Hodgkin lymphoma, *CHL-LR* lymphocyte-rich classical Hodgkin lymphoma, *CHL-LD* lymphocyte-depleted classical Hodgkin lymphoma, *NLPHL* nodular lymphocyte predominant Hodgkin lymphoma, # not available

The biopsy site of HL in children and adolescent was assessed in this study. Most HL occurred in the lymph node (Table 2). Extranodal site involvement in HL was uncommon (Table 2). 59.6% of NHL presented an extranodal site involvement at the time of diagnosis. The most common extranodal site of NHL involved the gastrointestinal tract (GI) (32.8%), skin (17.2%) and areas of the head and neck (16.2%) (Table 3).

**Table 2:** Biopsy site of HL in children and adolescents (*Ν* = 161)

Biopsy site	No.of cases
Lymph node	145
Mediastinum	11
Abdominal cavity	2
Uncertain	3

**Table 3:** Extranodal involvement of NHL in children and adolescents

Extranodal sites	% of extranodal NHLs
GI tract	32.8
Skin	17.2
Head and neck area	16.2
Mediastinum and lung	13.4
Bone	12.4
Reproductive system	3.5
CNS	1.3
Others	3.2

### Subtype distribution of lymphoma in children and adolescents

The majority (501/662, 75.7%) of lymphoma were NHL (Additional file 1: Table S1). One hundred sixty one cases (24.3%) were HL (Table 1). Of the NHL, the proportion of precursor cell lymphomas (TLBL/BLBL) was 32.0%, while the proportion of mature B-cell lymphomas and peripheral T/NK-cell lymphomas was 41.1% and 26.9%, respectively. The five common NHLs observed in children and adolescents were LBL (32.0%), BL (21.0%), ALCL (14.2%), DLBCL (13.8%) and ENKTCL (6.2%).

### EBV infection status of lymphoma in children and adolescents

**Fig. 1:**
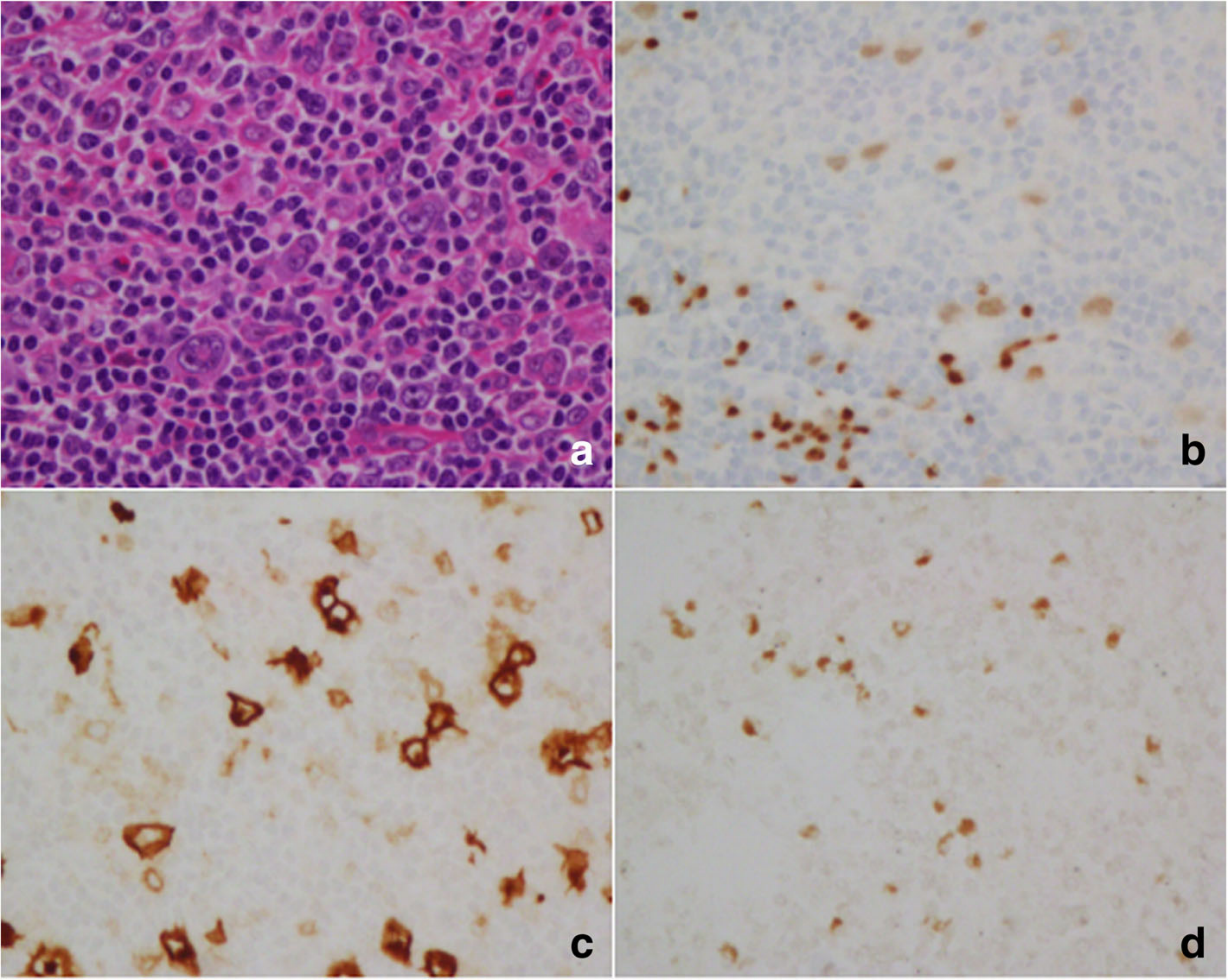
EBV infection in CHL (NS type) in children and young adolescents. **a** Mononuclear Hodgkin cells and mutinucleated Reed-Sternberg cells are seen in a cellular background rich in lymphocytes and some eosinophils (H&E, 400x). The neoplastic cells are positive for PAX-5 (**b**), CD30 (**c**) and EBERS in situ hybridization (**d**)

**Fig. 2:**
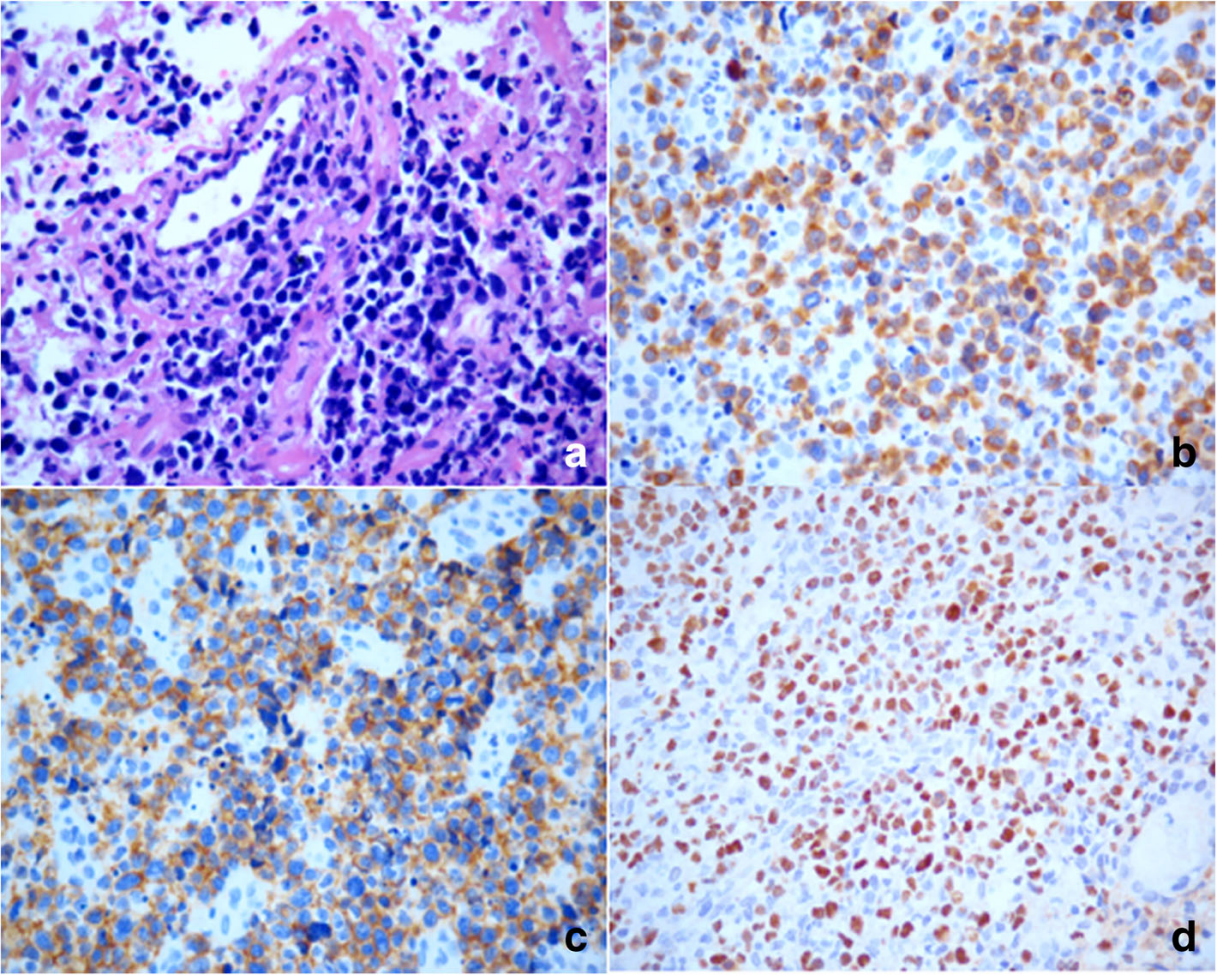
EBV infection in ENKTCL in children and young adolescents. **a** Medium to large-sized cells with pale cytoplasm (H&E, 400x). The neoplastic cells show strong staining for CD3 (**b**), CD56 (**c**) and EBERS in situ hybridization (**d**)

**Fig. 3:**
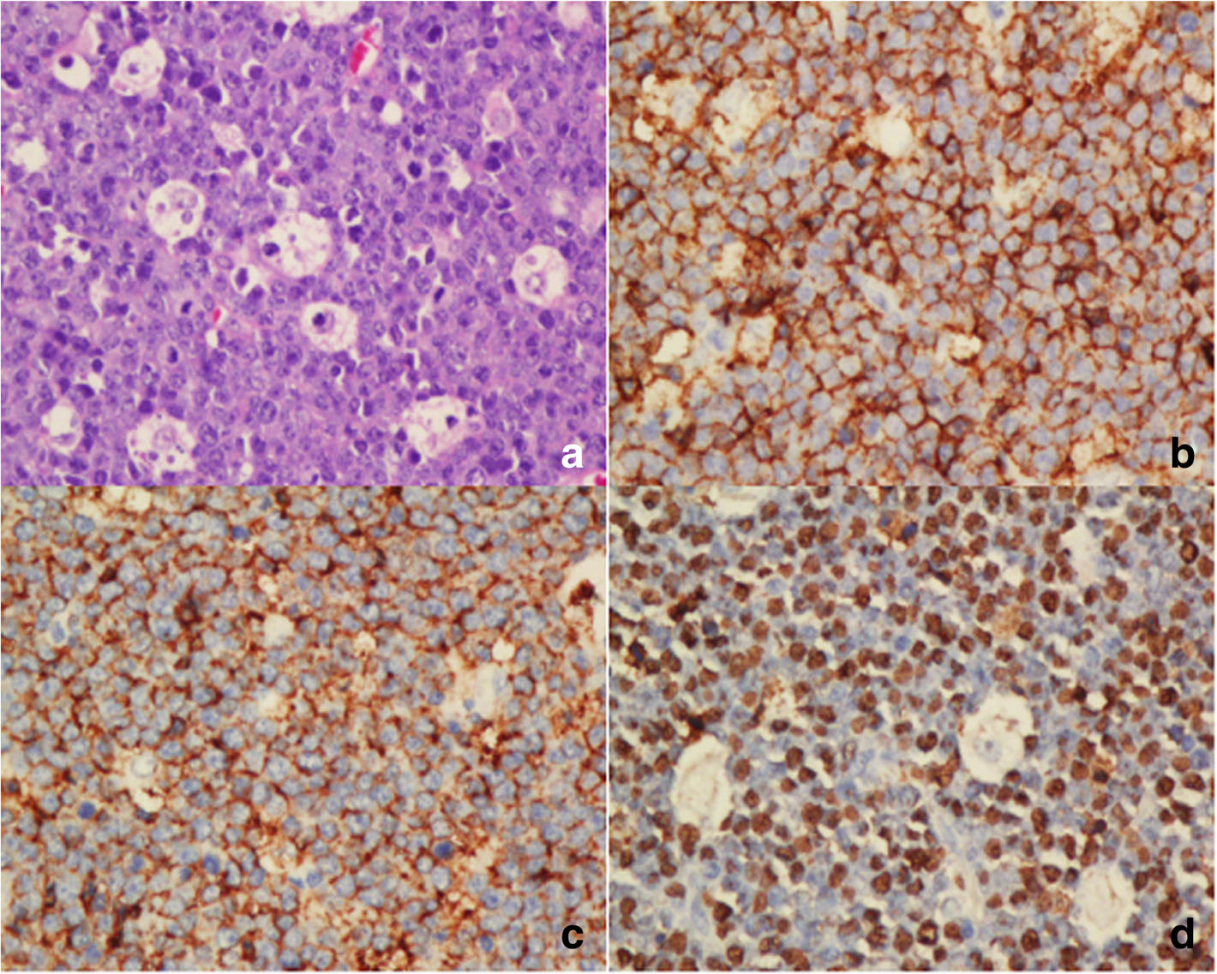
EBV infection in BL in children and young adolescents. **a** Uniform tumour cells with multiple small nucleoli and finely dispersed chromatin. A so-called starry sky pattern is presented (H&E, 400x). Immunohistochemistry shows strong positivity for CD20 (**b**), CD10 (**c**) and EBERS in situ hybridization (**d**)

**Fig. 4:**
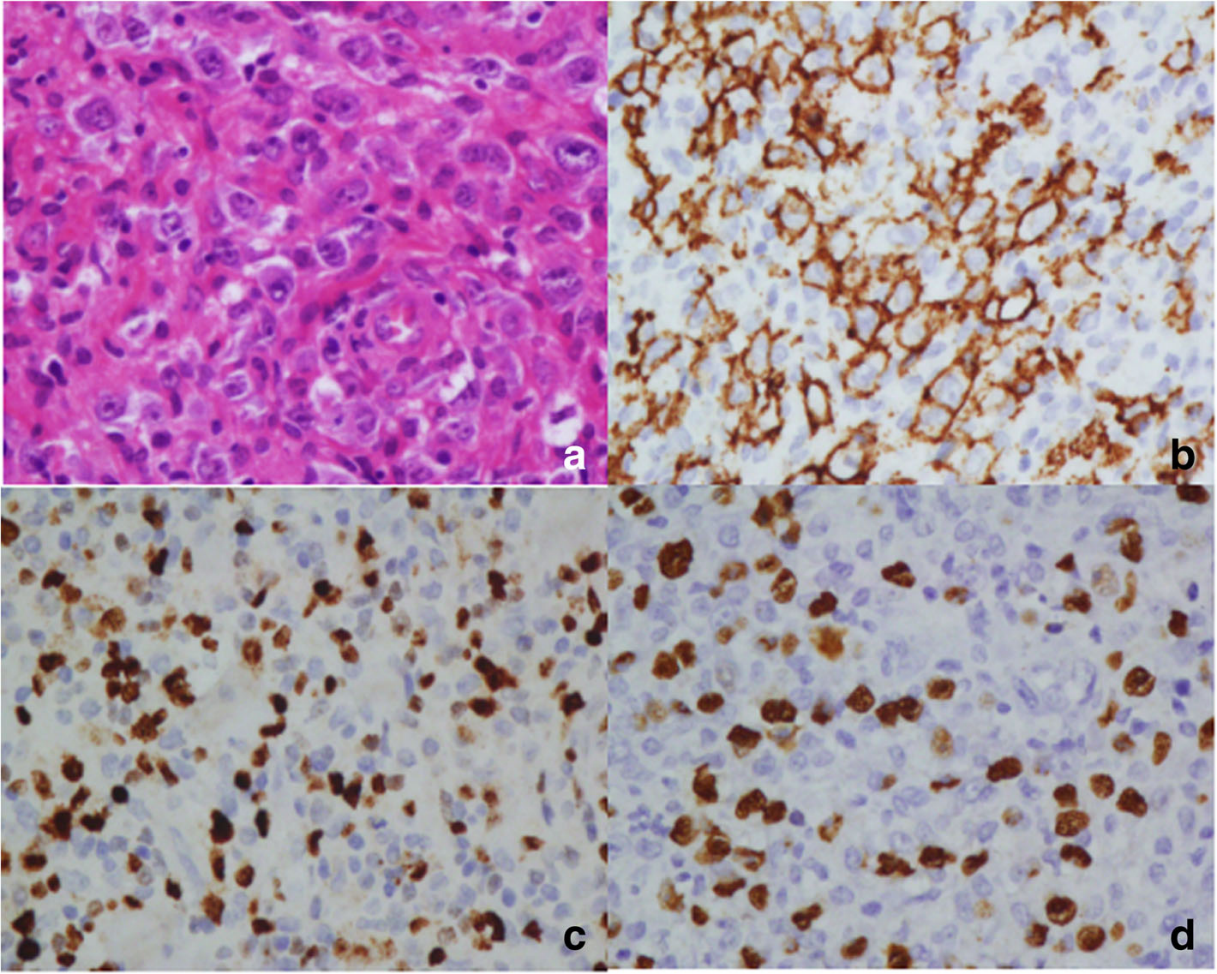
EBV infection in DLBCL in children and young adolescents. **a** Scattered large tumor cells are observed in a lymphohistiocytic microenvironment (H&E, 400x). Large neoplastic cells are positive for CD20 (**b**), Ki-67 (**c**) and EBERS in situ hybridization (**d**)

Ninety seven HLs were detected by EBERs in situ hybridization in this study, including 90 cases of CHL and 7 cases of Nodular lymphocyte predominant Hodgkin lymphoma (NLPHL). 58.9% cases of CHL (53/90) were EBV positive. No cases (0/7) of NLPHL were positive for EBERs. Among CHLs, mixed cellularity classical Hodgkin lymphoma (MC-CHL) showed the highest rate of positivity for EBERs (20/26, 76.9%), followed by Lymphocyte-rich classical Hodgkin lymphoma (LR-CHL) (11/16, 68.8%) (Table 1, Fig. 1). We also detected EBV in 256 NHLs, including 60 cases of LBL, 112 cases of mature B-cell lymphomas as well as 84 cases of peripheral T/NK-cell lymphomas. EBV infection was detected in 21.4% of mature B-cell lymphomas and 52.4% of peripheral T/NK-cell lymphomas. Hydroa vacciniforme-like lymphoproliferative disease (HVLLPD) also showed a strong association with EBV infection (11/11, 100%) (Additional file 1: Table S1). EBV was associated with high grade NHL including ENKTCL (100.0%), BL (30.5%) and DLBCL (17.6%) (Table 4, Fig. 2, Fig. 3, Fig. 4). No EBV infection was detected in ALCL (0/28). All lymphoblastic lymphomas were negative for EBERs (0/60).

**Table 4:** EBV infection status of lymphoma in children and adolescents in South China

Lymphoma Subtype	% of EBV+	
	In South China	In western countries
CHL	58.9	< 40.0 [25, 26]
NLPHL	0.0	~ 0.0 [27]
BL	30.5	15.0~ 30.0 [31]
DLBCL	17.6	3.0~ 15.0^[a]^ [36, 37]
ENKTCL	100.0	100.0 [28, 29]

^a^At present there are no large-scale epidemiologic data on EBV positive DLBCL in children and adolescents. We used the data in the elderly (> 50 years) from Asian, Latin American and western countries

## Discussion

Lymphoid neoplasms represent the dominant type of malignancy in children and adolescents worldwide, with the distribution of subtypes varying geographically. Major progression has been made in understanding the pathological characteristics of lymphoid neoplasms in the last 5 years, hence the WHO’s expanded spectrum of EBV related lymphomas [9, 10].

The population of South China is believed to have been be exposed to, or have a genetic susceptibility to EBV [13]. In this study, the clinicopathological features and EBV infection status of 662 cases of children and adolescents with lymphoma in South China were reviewed and analyzed.

The proportion of HL in children and adolescents in this study is 24.3% higher than that observed in western countries (11%-15%) [14]. Most HL patients showed lymphadenopathy which coordinates with other published data [15]. However, the majority of children and adolescents’ lymphomas was NHL. More than half of NHL (59.6%) patients were presented with extranodal site involvement at diagnosis which might make it difficult to detect the disease at an earlier stage [6].

Distribution of lymphoma subtypes is presented in this report. Compared to adult lymphoma MC-CHL has been reported as the most common subtype of CHL [2], in this study of children and adolescents, nodular sclerosis type CHL (NS-CHL) was more frequent than other subtypes of CHL, which is consistent with reports from other areas [7]. Peripheral T/NK cell lymphomas comprised of 26.9% of NHLs in South China, which is higher than that observed in western countries (15%-20%) [11]. LBL has been reported as the most common lymphoid neoplasms in children and adolescents (32.0% of NHLs in this study) with the dominant type of TLBL (70.0% in this study) [16]. BL accounted for 21.0% of NHLs, similar to other reports in western countries (20.0%-40.0%) [17], but it occurred more frequently compared to other areas of Asia (9-12%) [6]. The incidence of anaplastic large-cell lymphoma (ALCL) differs significantly worldwide (2%-20%) [6, 18]. In this study, ALCL was the third most common NHL in children and adolescents. Moreover, ENKTCL accounted for 22.9% of peripheral T/NK-cell lymphomas, indicating its prevalence in areas of East Asia [10, 19, 20]. HVLLPD, the nomenclature and definition changed from hydroa vacciniforme-like lymphoma (HVLL) to lymphoproliferative disorder due to its relationship with chronic active EBV infection and clinical course, comprised 8.1% of mature T/NK-cell neoplasms in this study. HVLLPD is extremely rare in western countries. Recurrent skin lesions with T-cell or NK-cell type in children and adolescents from Asian and Latin American countries have been described [11, 21]. Our data suggest CAEBV of T/NK-cell, perhaps persists only in genetically predisposed individuals.

Significant advancement in recent years has been made in better understanding MYC alterations of DLBCL [22] and the updated WHO classification of high grade B-cell lymphoma [11, 23]. Clinically, DLBCL in young patients is very aggressive and commonly treated with similar regimens to BL [24]. The proportion of this kind of lymphoma in our research is 13.8% which is similar to results found in the literature [16].

EBV is a ubiquitous lymphotropic gammaherpesvirus that infects > 90% of the world population, usually without adverse health consequences. However, EBV is associated with malignancies of B-cell origin, NK/T-cell origin, and epithelial malignancies, such as endemic BL, ENKTCL, HL and nasopharyngeal carcinoma (NPC).

The pooled prevalence of EBV infection in CHLs of children and adolescents was 58.9% in this study which was higher than that reported in western countries (fewer than 40%) [25, 26]. Specifically, EBV infection was detected in MC-CHL (76.9%), followed by lymphocyte-rich LR-CHL (75.0%) and NS-CHL type (44.7%). EBV infection showed no association with NLPHL which is in accordance with other reports [27].

The role of EBV in the pathogenesis of NHL has been observed in this study, 52.4% of peripheral T/NK-cell lymphomas and 21.4% of mature B-cell lymphomas were EBV positive. Moreover, EBV was associated with high grade NHL including ENKTCL, BL and DLBCL. No EBV was detected in LBL or ALCL.

It has been consistently documented that ENKTCL is associated with EBV infection [28, 29]. Other risk factors and the pathogenesis of ENKTCL are not well understood. Recently, a major breakthrough in the field of genetic research for ENKTCL has been found from southern China. The study shows that the susceptibility gene of ENKTCL is the HLA-DPB1 gene, suggesting that ENKTCL have distinct pathogenic mechanisms with NPC or HL [30].

The possible contribution of EBV to BL pathogenesis is mainly unknown. In one hypothesis, it has been suggested that EBV may be associated with all of the cases by a mechanism of hit-and-run [31]. Early during oncogenesis, viral genes potentiate tumor development. Progressively, viral genome is lost to escape the immune system, and proto-oncogene mutations promote tumor progression. The expression of EBV-encoded miRNAs by miRNA profiling can be observed in BL case including EBERs negative cases [32]. The “hit and run” model could explain why some cases of endemic BL are negative for latent EBV genomes.

EBV can be detected in more than 95% of endemic BL cases and approximately 20-30% of sporadic BL by EBERs. Molecular profiles and significant pathways of endemic BL and sporadic BL are different [33]. Endemic and HIV-related BL cases may derive from a later developmental stage of B cells, i.e. post-germinal centre/memory B cells [34]. In sporadic BL, the intrinsic activation of BCR pathway due to the mutations of TCF3/ID3 genes may further allow neoplastic cells to grow with or without EBV [35].

EBV related BL is more prevalent in this study (30.5%) compared to that of western countries [36]. The higher prevalence of EBV positive BL cases in children and adolescents in South China might result from early EBV infection [37].

EBV-positive DLBCL has been observed in younger patients and led to substituting the modified “elderly” with “not otherwise specified” (EBV+ DLBCL, NOS) in the updated classification [38]. The incidence of EBV infection among DLBCL from Asian or Latin American patients (elderly) ranges from 8% to 15%, less than 5% in western countries [39]. Our study showes the incidence of EBV infection in DLBCL in children and adolescents was 17.6%. Yet, further studies are needed to explore whether this incidence is different from that of adults in South China.

## Conclusions

The high proportion of peripheral T/NK-cell lymphomas in children and adolescents in South China is presented in this study and compared to western countries due to the high percentage of ENKTCL. ENKTCL is firmly associated with EBV infection, while more than half of HL, a portion of BL and DLBCL are related to EBV infection. This study conclusively demonstrates that EBV infection is more prevalent in children and adolescents with lymphomas in South China compared to western countries.

## Additional file

Additional file 1: **Table S1.** Distribution of NHL subtypes in children and adolescents in South China (*Ν* = 501). (DOCX 19 kb)

## Abbreviations

ALCL: Anaplastic large-cell lymphoma
ALK: Anaplastic large cell lymphoma kinase
BL: Burkitt lymphoma
CHL: Classical Hodgkin lymphoma
CHL-LD: Lymphocyte-depleted classical Hodgkin lymphoma
CHL-LR: Lymphocyte-rich classical Hodgkin lymphoma
CHL-MC: Mixed cellularity classical Hodgkin lymphoma
CHL-NS: Nodular sclerosis classical Hodgkin lymphoma
CT: Computed tomography
DLBCL: Diffuse large B cell lymphoma
EBERS: In situ hybridization for Epstein-Barr virus encoded RNAs
EBV: Epstein Barr virus
ENKTCL: Extranodal NK/T-cell lymphoma, nasal type
HL: Hodgkin lymphoma
HVLLPD: Hydro vaceinifomle-like cutaneous T-cell lymphoproliferative disorders/lymphoma
NHL: Non-Hodgkin lymphoma
NLPHL: Nodular lymphocyte predominant Hodgkin lymphoma
NPC: Nasopharyngeal carcinoma
TLBL/BLBL: Lymphoblastic lymphoma (precursor T-and precursor B-cell lymphoma
WHO: The World Health Organization
