# Supplementary material for: Clinicopathological features and EBV infection status of lymphoma in children and adolescents in South China: a retrospective study of 662 cases
Source: Diagn Pathol. 2018 Feb 27;13:17. doi: 10.1186/s13000-018-0693-0 (PMC5828429; doi:10.1186/s13000-018-0693-0)
Supplement: Supplementary file 1 — Table S1. Distribution of NHL subtypes in children and adolescents in South China (Ν = 501). (DOCX 19 kb) [file 13000_2018_693_MOESM1_ESM.docx]

| **Supplementary file 1: Distribution of NHL subtypes in children and adolescents in South China (Ν = 501)** | | | | | | | |
| --- | --- | --- | --- | --- | --- | --- | --- |
| Lymphoid Neoplasms | No.of cases | % of NHL | M:F Ratio | % of Extranodal site | % of EBERs + | |  |
| BLBL | 48 | 9.6 | 1.3 | 27.1 | | 0.0 (0/18 ) |  |
| TLBL | 112 | 22.4 | 0.9 | 61.6 | | 0.0 (0/42) |  |
| **Mature B-cell**  **Neoplasms** | 206 | 41.1 | 2.7 | 66.5 | | 21.4 (24/112) |  |
| BL | 105 | 21.0 | 5.6 | 70.5 | | 30.5 (18/59) |  |
| DLBCL | 69 | 13.8 | 1.5 | 60.3 | | 17.6 (6/34) |  |
| PFL | 12 | 2.4 | 3.0 | 33.3 | | 0.0 (0/9) |  |
| PMBL | 11 | 2.2 | 1.2 | 0.0 | | 0.0 (0/2) |  |
| MALT | 4 | 0.8 | # | 100.0 | | 0.0 (0/4) |  |
| FL | 3 | 0.6 | # | 0.0 | | 0.0 (0/3) |  |
| PL | 2 | 0.4 | # | 100.0 | | 0.0 (0/1) |  |
| **Peripheral T/NK-cellNeoplasms** | 135 | 26.9 | 1.8 | 60.7 | | 52.4 (44/84) |  |
| ALCL | 71 | 14.2 | 1.8 | 36.6 | | 0.0 (0/28) |  |
| ENKTCL | 31 | 6.2 | 0.8 | 96.8 | | 100.0 (27/27) |  |
| HVLLPD | 11 | 2.2 | 0.8 | 100.0 | | 100.0 (11/11) |  |
| Systemic EBV+ TL of Childhood | 6 | 1.2 | # | 50.0 | | 100.0 (6/6) |  |
| SPTCL | 6 | 1.2 | # | 100.0 | | 0.0 (0/4) |  |
| PTCL,NOS | 5 | 1.0 | # | 40.0 | | 0.0 (0/5) |  |
| PCCD30+LPD | 3 | 0.6 | # | 100.0 | | 0.0 (0/1) |  |
| MF | 1 | 0.2 | # | 100.0 | | 0.0 (0/1) |  |
| CGD-TCL | 1 | 0.2 | # | 0.0 | | 0.0 (0/1) |  |

ALCL, anaplastic large-cell lymphoma; DLBCL, diffuse large B-cell lymphoma; ENKTCL, extranodal NK/T-cell lymphoma, nasal type; MALT, extranodal marginal zone lymphoma of mucosa associated lymphoid tissue; FL, follicular lymphoma; LBL, lymphoblastic leukemia/lymphoma; PTCL,NOS, peripheral T-cell lymphoma, not otherwise specified; MCL, mantle cell lymphoma; BL, Burkitt lymphoma; SPTCL, subcutaneous panniculitis-like T-cell lymphoma; Systemic EBV+ TL of childhood, systemic EBV-positive T-cell lymphoma of childhood; CGD-TCL; cutaneous gamma/delta T-cell lymphoma; PCCD30+LPD, primary cutaneous CD30 positive lymphoproliferative disorders; MF, mycosis fungoides; PL, plasma cell neoplasms; HVLLPD, hydro vaceinifomle-like lymphoproliferative disorders; #, not available.
